# Supplementary figures and images for: Wellbeing across the American Nations: First Settler Effects influence traditional and existential wellness
Source: PLoS One. 2025 Sep 10;20(9):e0327972. doi: 10.1371/journal.pone.0327972 (PMC12422463; doi:10.1371/journal.pone.0327972)

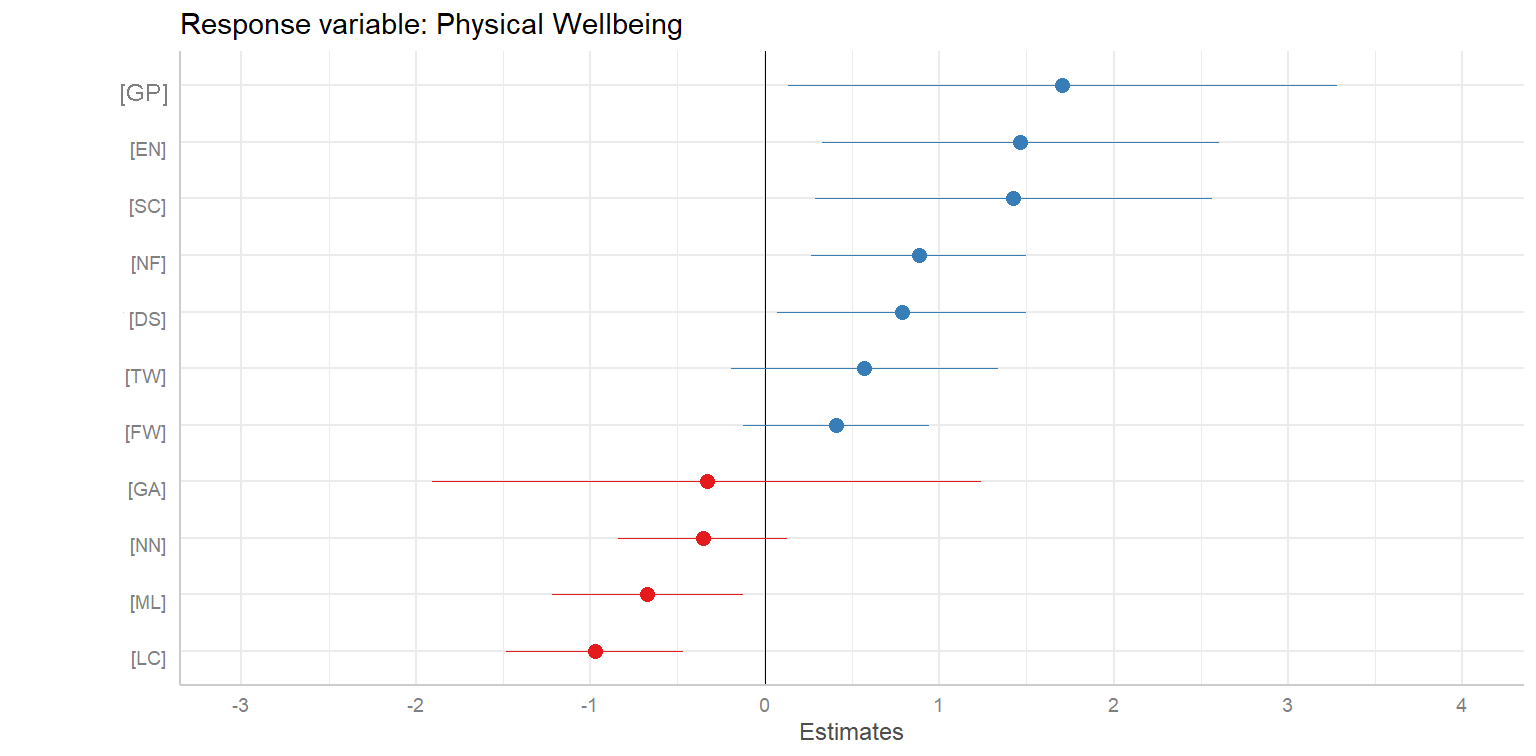

Supplement: S1 Fig — Summary plot of physical wellbeing scores across MSAs in the U.S. (TIF) [file pone.0327972.s001.tif]

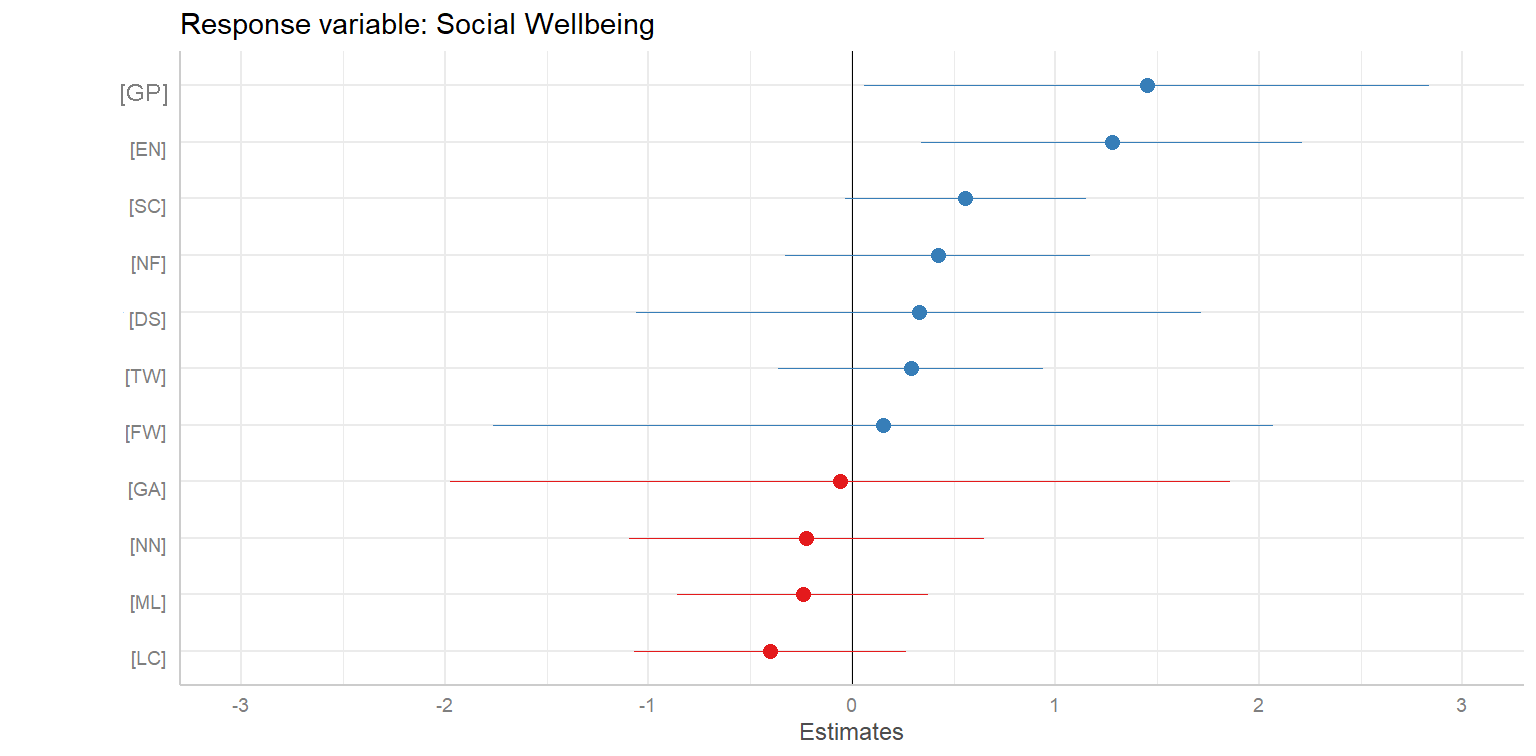

Supplement: S2 Fig — Summary plot of social wellbeing scores across MSAs in the U.S. (TIF) [file pone.0327972.s002.tif]

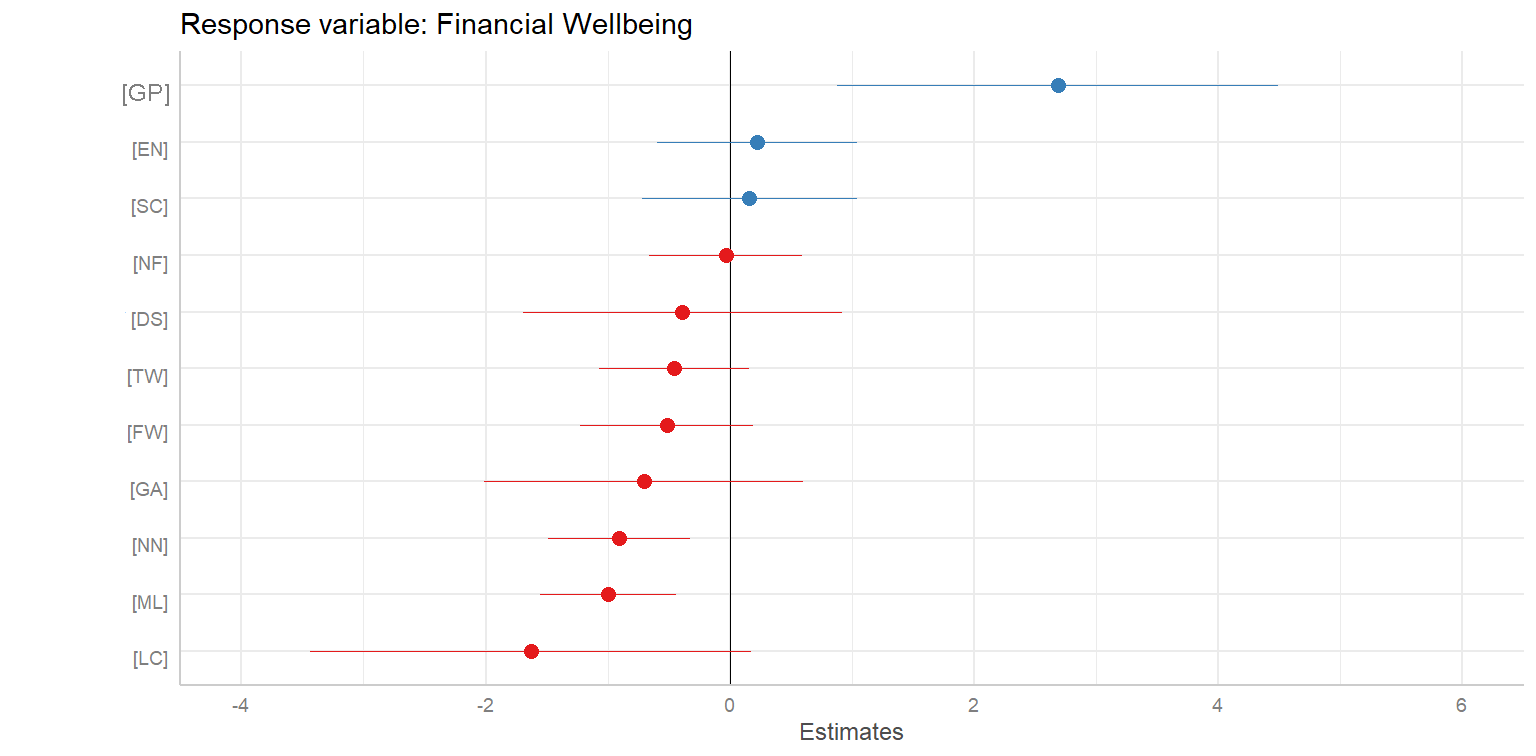

Supplement: S3 Fig — Summary plot of financial wellbeing scores across MSAs in the U.S. (TIF) [file pone.0327972.s003.tif]

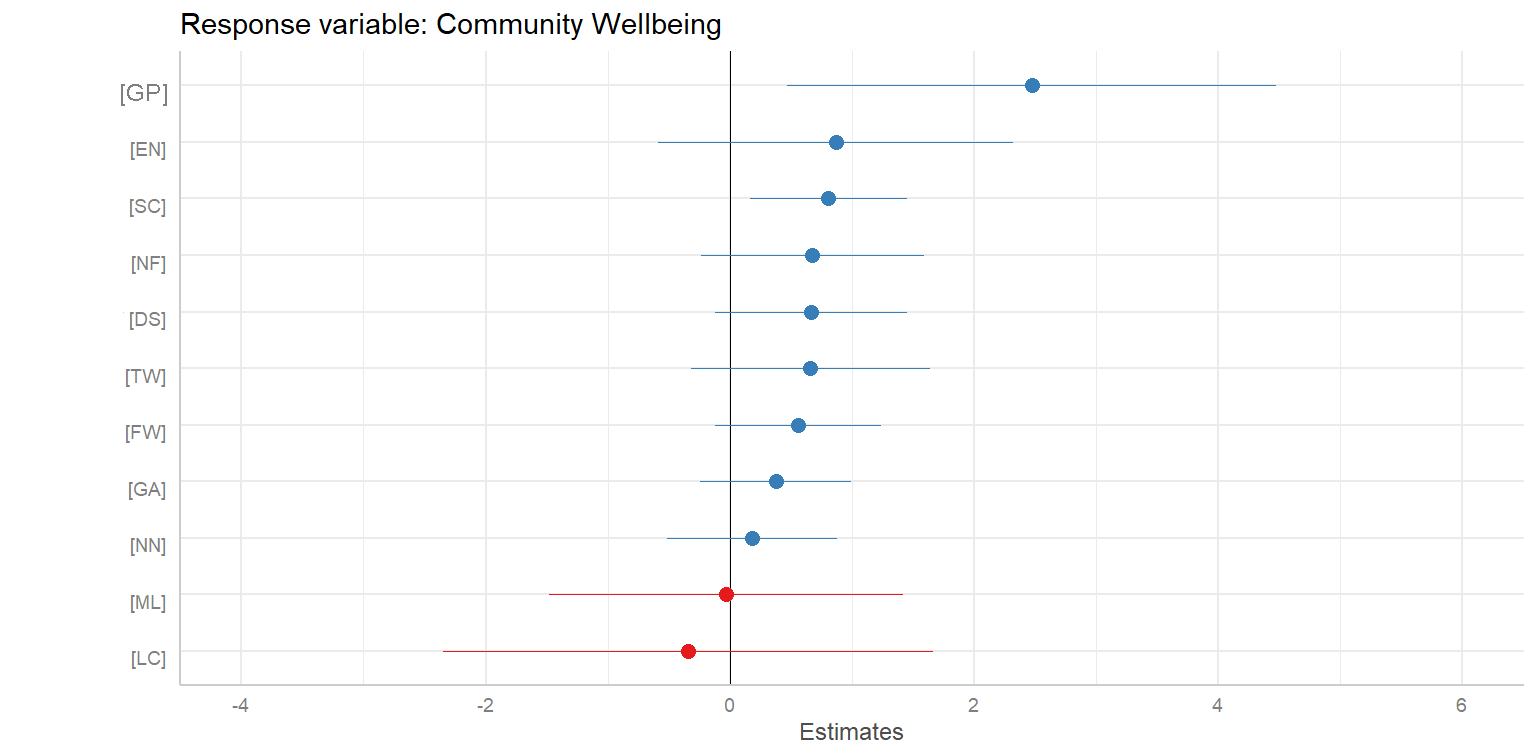

Supplement: S4 Fig — Summary plot of community wellbeing scores across MSAs in the U.S. (TIF) [file pone.0327972.s004.tif]

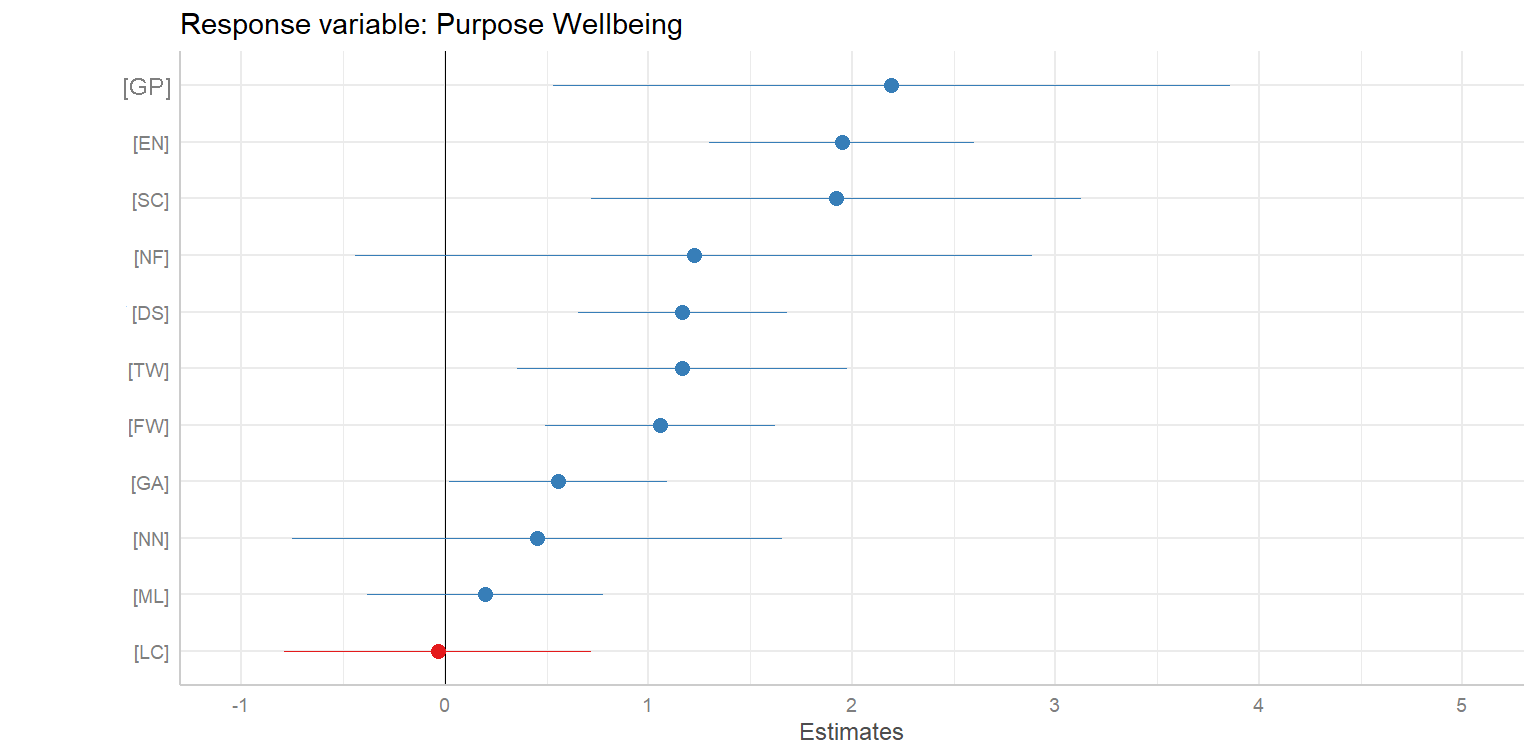

Supplement: S5 Fig — Summary plot of purpose wellbeing scores across MSAs in the U.S. (TIF) [file pone.0327972.s005.tif]
