# Supplementary material for: Wellbeing across the American Nations: First Settler Effects influence traditional and existential wellness
Source: PLoS One. 2025 Sep 10;20(9):e0327972. doi: 10.1371/journal.pone.0327972 (PMC12422463; doi:10.1371/journal.pone.0327972)
Supplement: Attachment — Submitted filename: Wellbeing - First Nations - Review.pdf [file pone.0327972.s006.pdf]

Dear Authors,

Thank you for writing such a fascinating paper. Here, I believe this paper should be published, though it has a few major and minor issues that I believe should be addressed to substantially improve the manuscript.

Major Issues:

1. The manuscript repeatedly references the $75,000 income plateau in well-being (Kahneman & Deaton, 2010). However, more recent research by Killingsworth (2021), which used real-time, continuous measures of experienced well-being in a large U.S. sample, found a linear relationship with income across the full range, including well above $75,000. A revised introduction/discussion should acknowledge this recent research and discuss the nuanced relationship between material wealth and wellbeing accordingly.
2. The manuscript introduces the First Settler Wellness Effect and asserts that early settlement patterns explain current wellness differences, but it does not sufficiently elaborate the psychological mechanisms underlying this persistence. Specifically, the psychological or institutional processes through which regional cultures continue to influence wellness outcomes today, despite changes in ecology, economy, and demography, remain vague. For example, while honor cultures are attributed to herding societies, there is no explanation of why those patterns persist now that herding has largely declined. Integrating theories of cultural transmission would help clarify and tighten the hypotheses for how the historical legacies hypothesized translate into contemporary individual-level outcomes.
3. The classification of social well-being within the Traditional Wellness Index, rather than the Existential one, needs further justification. Social relationships seem quite related to existential wellness. Did the authors empirically test the factor structure of the wellness components to examine their validity? Does social well-being fit better in the traditional wellness index? Demonstrating this would be useful.
4. The manuscript implies plausible psychological and social mechanisms through which regional culture influences different dimensions of wellness, but these are not formally tested. In particular, two mediation models seem especially warranted: (1) Region → Social Connection → Existential Wellness, which reflects how communal norms and belonging foster purpose and meaning; and (2) Region → Institutional Trust/Strength → Traditional Wellness, which reflects how confidence in institutions (e.g., health care, governance, economic systems) supports material and physical stability. Testing these pathways empirically, through mediation models, would clarify the mechanisms of the First Settler Wellness Effect and strengthen the claims.

5. Given the conceptual distinction between traditional and existential wellness, it would be informative to test whether the predictors have significantly different effects across these two wellness types. This could be accomplished via a within-subjects mixed-effects model that includes a wellness-type factor and its interactions with the primary predictors. Such an analysis could more directly assess claims regarding the differences or tradeoffs between material stability and existential fulfillment across regions. That is, do the relationships between regions and each wellness type significantly differ from one another?

Minor issues:

6. The manuscript presents the First Settler Effects hypothesis and the American Nations framework in a way that feels overly deterministic. While the model offers a useful lens, it has also been subject to scholarly critique for its generalizations. A brief acknowledgment of these critiques in the introduction would help temper the stronger claims and reflect the diversity within regions in the U.S.
7. The manuscript states that "additional research has found a significant association between having a strong internal locus of control and overall happiness," but no citation is provided for this claim.
8. While the study's central findings are compelling and well-supported, the final discussion section would benefit from substantial tightening. Much of the latter half reads as exploratory rather than integrative, with several tangents (e.g., early childhood education, DRAMMA framework, internet use, indigenous communities) that, although interesting, dilute its main focus.
